# Inactivation of Endothelial ADAM17 Reduces Retinal Ischemia-Reperfusion Induced Neuronal and Vascular Damage

**DOI:** 10.3390/ijms21155379

**Published:** 2020-07-29

**Authors:** Diana R Gutsaeva, Lamiaa Shalaby, Folami L Powell, Menaka C Thounaojam, Hossameldin Abouhish, Sara A Wetzstein, Ravirajsinh N Jadeja, Hang Fai Kwok, Pamela M Martin, Manuela Bartoli

**Affiliations:** 1 Department of Ophthalmology, Medical College of Georgia, Augusta University, Augusta, GA 30912, USA; LSHALABY@augusta.edu (L.S.); mthounaojam@augusta.edu (M.C.T.); habouhish@augusta.edu (H.A.); mbartoli@augusta.edu (M.B.); 2 Department of Biochemistry and Molecular Biology, Medical College of Georgia, Augusta University, Augusta, GA 30912, USA; FPOWELL@augusta.edu (F.L.P.); rjadeja@augusta.edu (R.N.J.); pmmartin@augusta.edu (P.M.M.); 3 Department of Clinical Pharmacology, Faculty of Medicine, Mansoura University, 35516 Mansoura, Egypt; 4 School of Medicine, Mercer University, Macon, GA 31207, USA; Sara.Ann.Wetzstein@live.mercer.edu; 5 Institute of Translational Medicine, Faculty of Health Sciences, University of Macau, Avenida de Universidade, Macau 999078; hfkwok@um.edu.mo

**Keywords:** retinal ischemia-reperfusion, ADAM17, vascular permeability, neuronal and vascular degeneration

## Abstract

Retinal ischemia contributes to visual impairment in ischemic retinopathies. A disintegrin and metalloproteinase ADAM17 is implicated in multiple vascular pathologies through its ability to regulate inflammatory signaling via ectodomain shedding. We investigated the role of endothelial ADAM17 in neuronal and vascular degeneration associated with retinal ischemia reperfusion (IR) injury using mice with conditional inactivation of ADAM17 in vascular endothelium. ADAM17Cre-flox and control ADAM17flox mice were subjected to 40 min of pressure-induced retinal ischemia, with the contralateral eye serving as control. Albumin extravasation and retinal leukostasis were evaluated 48 h after reperfusion. Retinal morphometric analysis was conducted 7 days after reperfusion. Degenerate capillaries were assessed by elastase digest and visual function was evaluated by optokinetic test 14 and 7 days following ischemia, respectively. Lack of ADAM17 decreased vascular leakage and reduced retinal thinning and ganglion cell loss in ADAM17Cre-flox mice. Further, ADAM17Cre-flox mice exhibited a remarkable reduction in capillary degeneration following IR. Decrease in neurovascular degeneration in ADAM17Cre-flox mice correlated with decreased activation of caspase-3 and was associated with reduction in oxidative stress and retinal leukostasis. In addition, knockdown of ADAM17 resulted in decreased cleavage of p75NTR, the process known to be associated with retinal cell apoptosis. A decline in visual acuity evidenced by decrease in spatial frequency threshold observed in ADAM17flox mice was partially restored in ADAM17-endothelial deficient mice. The obtained results provide evidence that endothelial ADAM17 is an important contributor to IR-induced neurovascular damage in the retina and suggest that interventions directed at regulating ADAM17 activity can be beneficial for alleviating the consequences of retinal ischemia.

## 1. Introduction

Retinal ischemia contributes to visual impairment in ischemic retinopathies, such as diabetic retinopathy and retinopathy of prematurity, and is associated with other ocular pathologies, such as retinal vascular occlusion and acute angle-closure glaucoma [1,2,3]. As in other vascular beds, the important component of the pathogenesis of retinal ischemia is tissue damage caused by transient ischemic insult, which is determined by the magnitude and duration of the interruption in blood supply, and then subsequent damage induced by tissue reperfusion [4]. The complete understanding of the mechanisms underlying vascular and neuronal cell damage in the ischemic retina is still lacking, thus preventing the development of effective therapeutic interventions.

Neuronal cell damage is a well-described phenomenon in retinal ischemia reperfusion (IR) injury and is consistently reproduced in rodent models of retinal IR. Previous studies have reported that IR results in decreased retinal function evidenced by a reduction of a-wave and b-wave amplitudes, a decline in optokinetic responses, and increased neuronal cell death [5,6,7,8]. Consistent with retinal neuronal cell loss, the IR-injured retina displays reduced thickness of the ganglion and nuclear cell layers [5,7]. More recently, several research groups reported that IR also recapitulates vascular impairments observed in the diabetic retina such as loss of vascular barrier function and capillary degeneration [9,10,11,12]. Notably, neuronal cell loss in the IR-injured retina precedes vascular capillary degeneration [9]. It was recognized that the excessive generation of reactive oxygen species (ROS) and vascular inflammation accompanying IR injury contribute to retinal vascular and neuronal cell death [9,13,14].

Proteolytic cleavage of extracellular domain by members of a disintegrin and metalloproteinase (ADAMs) family of proteins is an important posttranslational modification that can lead to activation, inhibition, or significant modulation of the function of cleaved proteins. ADAM17, a disintegrin and metalloproteinase 17, was first described as an enzyme promoting the release of soluble TNFα from its membrane-bound precursor [15] and, therefore, was extensively validated as a target in preclinical models for anti-TNFα therapy [16,17,18]. Later, it was documented that ADAM17 is implicated in proteolytic cleavage of many other mediators involved in inflammation, immune responses, and neurodegenerative disorders [19,20,21,22,23]. Among ADAM17 substrates are growth factors such as transforming growth factor (TGFα) and heparin-binding EGF-like growth factor (HB-EGF), cytokine receptors such as TNFR-I/II, interleukin-6 receptor (IL-6R), a neurotrophin receptor (p75NTR), adhesion molecules such as vascular cell adhesion molecule-1 (VCAM-1) and intercellular adhesion molecule-1 (ICAM-1), tight junctions proteins such as junction adhesion molecule-A (JAM-A), and chemokines such as CX3CL1 [15,24,25,26,27,28,29].

Accumulating data link over-activation of ADAM17 to diverse vascular pathologies such as thoracic and abdominal aortic aneurysm, pathophysiological vascular remodeling, age-related coronary microvascular dysfunction, and pathologic retinal neovascularization [30,31,32,33,34]. In our previous work, we uncovered the contributing role for endothelium-derived ADAM17 in promoting vascular alterations associated with early experimental diabetes [35]. In the present study, we sought to determine whether vascular ADAM17 also contributes to cellular injury in the ischemic retina. For this purpose, we used conditional knockout mice that lack expression of ADAM17 in endothelial cells.

## 2. Results

### 2.1. IR Injury Upregulates Expression and Activity of Retinal ADAM17

**Figure 1:**
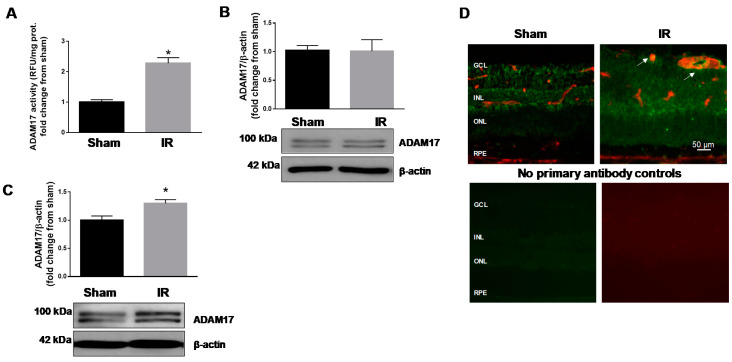
ADAM17 expression and enzymatic activity are increased in retinas of C57Bl/6J mice subjected to retinal IR (ischemia reperfusion). (**A**) ADAM17 enzymatic activity and (**B**) protein expression in IR-injured and sham-operated eyes of C57Bl/6J mice 6 h after IR were evaluated by fluorimetric assay and Western blotting analysis, respectively. Values are mean ± standard error (SE). Results are presented as a fold change from sham. * *p* < 0.001 vs. corresponding sham; n = 5 in each group. (**C**) Western blotting analysis of ADAM17 expression 48 h after reperfusion. Blots were subjected to densitometric analysis and the obtained data were analyzed for statistical significance. Actin was used as an internal control. Values are mean ± SE. Results are presented as a fold change from sham. * *p* < 0.05 vs. corresponding sham; *n* = 5 in each group. (**D**) Representative immunofluorescence images show expression of ADAM17 (green) in retinal vessels of IR-injured and sham-operated eyes 48 h after IR (white arrows). Retinal blood vessels were co-labeled with anti-CD31 antibody (red); *n* = 3 in each group. Scale bar, 50 µm. GCL, ganglion cell layer; INL, inner nuclear layer; ONL, outer nuclear layer; RPE, retinal pigment epithelium.

We previously showed that hyperglycemia results in upregulation of retinal ADAM17 in human and experimental diabetes [35]. To verify whether ADAM17 is upregulated in retinal IR model, we measured expression and enzymatic activity of this sheddase in C57Bl/6J mice subjected to ischemia followed by different periods of reperfusion. Our data showed that 40 min of ischemia followed by 6 h of reperfusion caused an increase in enzymatic activity of ADAM17 (Figure 1A). No difference in ADAM17 protein expression in the IR-injured retina was observed at this time point (Figure 1B). The prolongation of the reperfusion time to 48 h also led to upregulation of ADAM17 at protein levels in the injured eye compared to the control sham-operated eye (Figure 1C). In addition, colocalization of ADAM17 with endothelial cell marker CD31 showed that IR (48 h) led to increased ADAM17 immunoreactivity in the retinal microvasculature of the ischemic eye as evidenced by increased yellow fluorescence signal in retinal cryosections (Figure 1D; white arrows).

**Figure 2:**
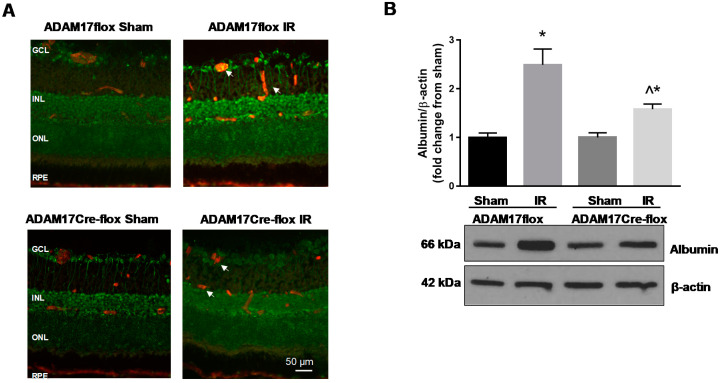
IR-induced vascular leakage is diminished in mice with conditional inactivation of ADAM17 in vascular endothelium. (**A**) Representative immunofluorescence images of ADAM17 expression (green) in retinas of control ADAM17flox and ADAM17Cre-flox mice following IR. Retinal blood vessels (white arrows) were co-labeled with anti-CD31 antibody (red); *n* = 3 in each group. Scale bar, 50 µm. (**B**) Western blotting analysis of extravascular albumin in retinal tissue of ADAM17flox and ADAM17Cre-flox mice was performed 48 h after IR. Blots were subjected to densitometric analysis and the obtained data were analyzed for statistical significance. Actin was used as an internal control. Values are mean ± SE. Results are presented as fold of change from sham. * *p* < 0.01 vs. corresponding sham; ^ *p* < 0.001 vs. ADAM17flox IR; *n* = 5 in each group.

To further investigate the specific contribution of endothelium-derived ADAM17 to retinal IR-induced injury, we used mice lacking ADAM17 expression in the vascular endothelium, as described before [35]. Control ADAM17flox mice and mice lacking endothelial expression of ADAM17 (ADAM17Cre-flox or ADAM17 k/o mice) were subjected to retinal IR. As shown in Figure 2A, minimal expression of ADAM17 was observed in retinal vasculature of sham-operated ADAM17flox mice. No expression of ADAM17 was present in retinal vessels of sham-operated ADAM17 k/o mice (Figure 2A). Ischemia followed by 48 h of reperfusion significantly upregulated expression of ADAM17 in control ADAM17flox mice but not in endothelial k/o ADAM17Cre-flox mice (Figure 2A; white arrows).

### 2.2. Knockdown of Endothelial ADAM17 Reduces Retinal Vascular Permeability after IR

Previously, we showed that knockdown of endothelial ADAM17 protected diabetic retinas from vascular leakage [35]. To determine whether upregulation of endothelial ADAM17 also contributes to vascular permeability in the retinal IR model, we measured extravasation of albumin into retinal tissue in control ADAM17flox and endothelial k/o mice 48 h after reperfusion. Our Western blotting analysis showed that in ischemia-injured eyes of control ADAM17flox mice, leakage of albumin was significantly increased compared to sham-operated eyes (~2.5 fold, *p* < 0.05; Figure 2B). Knockdown of endothelial ADAM17 substantially attenuated the effects of IR on vascular permeability, as demonstrated by decreased albumin extravasation in retinal tissue of ADAM17 k/o mice (*p* < 0.05 compared to ADAM17flox IR; Figure 2B). There was no difference in vascular leakage between ADAM17flox and ADAM17Cre-flox mice without exposure to IR (Figure 2B). These data suggest that endothelial ADAM17 activity contributes to the blood retinal barrier breakdown and vascular permeability in IR-injured retinas.

### 2.3. Deletion of ADAM17 in the Endothelium Reduces Retinal Thinning and Cell Loss in the Ganglion Cell Layer (GCL) after IR

**Figure 3:**
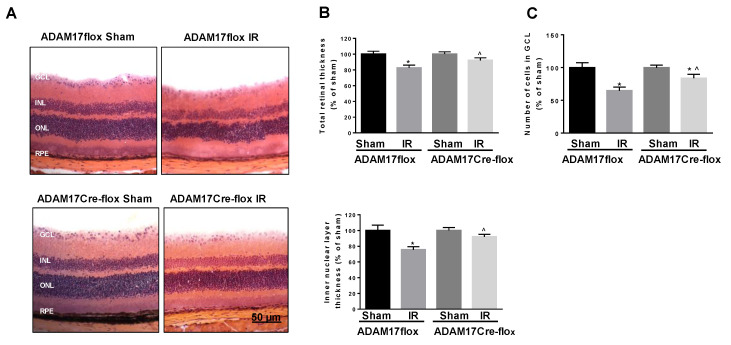
Endothelial ADAM17 is involved in IR-induced neuronal degeneration. (**A**) Representative hematoxylin and eosin stained retinal cryosections (mid-peripheral field) from IR-injured and sham-operated eyes of control ADAM17flox and ADAM17Cre-flox mice 7 days after ischemic insult. Scale bar, 50 µm. (**B**) Morphometric analysis of retinal cryosections: total retinal thickness and thickness of the INL. Data are expressed as mean ± SE and presented as a percent change from sham. * *p* < 0.01 vs. corresponding sham; ^ *p* < 0.05 vs. ADAM17flox IR; *n* = 5 for each group. (**C**) Quantification of cells in the GCL of IR-injured and sham-operated eyes of control ADAM17flox and ADAM17Cre-flox mice 7 days after ischemic insult. Data are expressed as cell number/100 μm retinal length and presented as a percent change from sham. * *p* < 0.001 vs. corresponding sham. ^ *p* < 0.0001 vs. ADAM17flox IR; *n* = 5 in each group.

**Figure 4:**
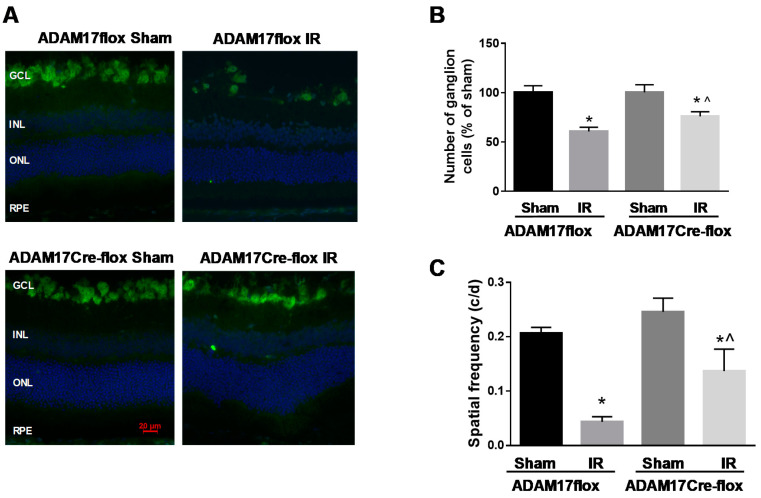
IR-induced ganglion cell loss is decreased in mice with conditional inactivation of endothelial ADAM17. (**A**) Representative immunofluorescence images of retinal cryosections from control ADAM17flox and ADAM17Cre-flox mice 7 days after ischemic insult identifying retinal ganglion cells (RBPMS-positive cells; green). Nuclei were stained with 4′,6-diamidino-2-phenylindole (DAPI; blue). Scale bar, 20 µm. (**B**) Quantification of RBPMS-positive cells. Data are expressed as cell number/100 μm retinal length and presented as a percent change from sham. * *p* < 0.01 vs. corresponding sham. ^ *p* < 0.05 vs. ADAM17flox IR; *n* = 5 in each group. (**C**) Visual acuity is improved in mice lacking endothelial ADAM17. Optokinetic tracking response was measured in control ADAM17flox and ADAM17Cre-flox mice subjected to IR using the OptoMotry system. Data are shown as mean ± SE. * *p* < 0.05 vs. corresponding sham; ^ *p* < 0.05 vs. ADAM17flox IR; *n* = 5 for each group.

IR leads to neuronal cell loss, which is accompanied by morphologic distortions and thinning of the retina cell layers [7,9]. We performed morphometric analysis of H&E (hematoxylin and eosin)- stained retinal sections from ADAM17flox and ADAM17Cre-flox mice 7 days after ischemia. As shown in Figure 3A,B, IR resulted in significant reduction in the total retinal thickness (~17% compared to sham; *p* < 0.001) and the thickness of the inner nuclear layer (INL) (~24% compared to sham; *p* < 0.01) in control ADAM17flox mice. Preservation of both the total retinal thickness (*p* < 0.05 compared to ADAM17flox IR) and the INL thickness (*p* < 0.001 compared to ADAM17flox IR) was observed in IR-injured eyes of conditional ADAM17 k/o mice (Figure 3A,B). Since degeneration of neuronal cells in the GCL (ganglion cell layer) is an important characteristic of the retinal damage following IR [7,9], we determined whether inactivation of endothelial ADAM17 affects cell loss in the GCL. A comparison of the cell number in the GCL in the sham-operated retinas showed no difference between of ADAM17flox and ADAM17Cre-flox mice. Following IR, the cell loss in the GCL of the ADAM17Cre-flox mice was significantly reduced as compared to control ADAM17flox mice (*p* < 0.05 compared to ADAM17flox IR; Figure 3C). Consistent with these data, further analysis of the number of ganglion cells, identified as cells immune-positive to anti-RNA binding protein with multiple splicing (RBPMS), in ADAM17flox and ADAM17Cre-flox mice revealed that lack of endothelial ADAM17 provided a partial protection to IR-injured retinas from loss of ganglion cells (~24% in ADAM17Cre-flox IR compared to ~40% in ADAM17flox IR; *p* < 0.05; Figure 4A,B).

### 2.4. Knockdown of Endothelial ADAM17 Reduces IR-Induced Loss in Visual Function

To assess whether knockdown of endothelial ADAM17 affects visual function, we employed a visual acuity test [36]. Using the virtual optokinetic system (CerebralMechanics, Lethbridge, AB, Canada), we measured optomotor reflex-based spatial frequency threshold in IR-injured (7 days of reperfusion) and control sham-operated ADAM17flox and ADAM17Cre-flox mice. No difference in spatial frequency threshold was observed between sham-operated eyes of ADAM17flox and endothelial ADAM17-deficient mice (0.21 ± 0.011 and 0.25 ± 0.025 for ADAM17flox and ADAM17Cre-flox, respectively; Figure 4C). IR caused a marked decrease in visual function in ADAM17flox mice as demonstrated by reduction in spatial frequency threshold (*p* < 0.001 compared to corresponding sham; Figure 4C). Ablation of endothelial ADAM17 partially recovered visual function as evidenced by the increase in spatial frequency threshold in IR-injured eyes in ADAM17Cre-flox mice (*p* < 0.05 compared to ADAM17flox IR; Figure 4C). These results are consistent with our morphologic analysis showing partial recovery from neuronal cell loss in mice lacking ADAM17.

### 2.5. Inactivation of Endothelial ADAM17 Decreases a Number of Degenerate Capillaries Following IR

**Figure 5:**
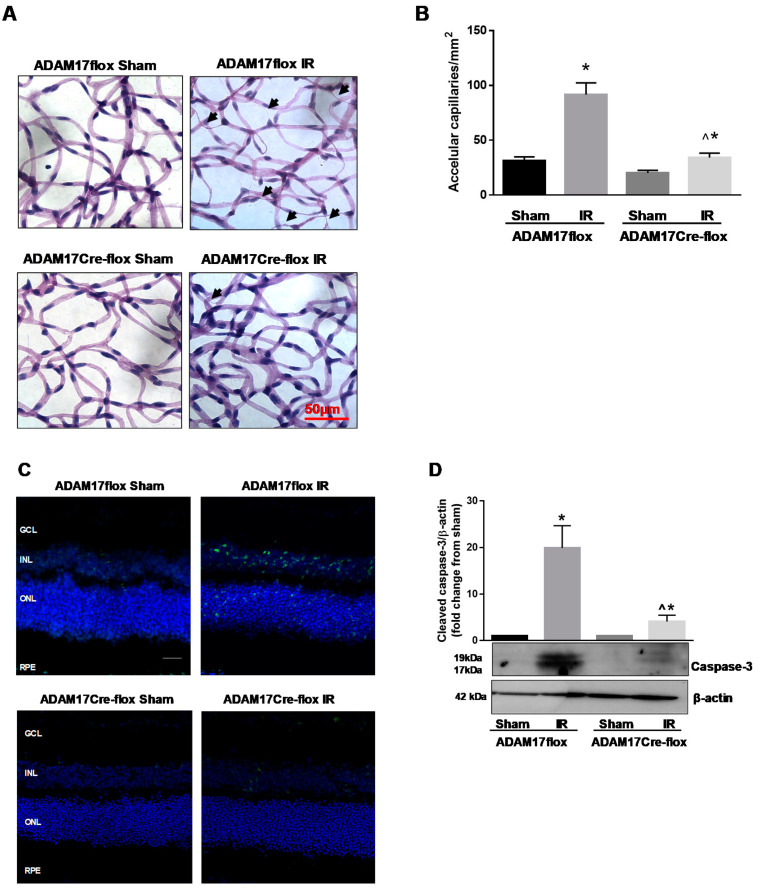
IR-induced vascular degeneration is reduced in mice with genetic ablation of endothelial ADAM17. (**A**) Representative images of retinal vascular digest and (**B**) quantification of degenerate capillaries in control ADAM17flox and ADAM17Cre-flox mice subjected to retinal IR. Arrows point at degenerate capillaries. Values are mean ± SE. * *p* < 0.05 vs. corresponding sham; ^ *p* < 0.001 vs. ADAM17flox IR; *n* = 5 for each group. Scale bar, 50 µm. (**C**) TUNEL (terminal deoxynucleotidyl transferase dUTP nick end labeling) staining (green) to assess apoptosis in retinal cryosections 48 h after reperfusion. Nuclei were stained with DAPI (blue). Scale bar, 20 µm. (**D**) Representative Western blots and densitometric analysis of cleaved caspase-3 evaluated 48 h after reperfusion. Actin was used as an internal control. Values are mean ± SE. Results are presented as fold change from sham. * *p* < 0.05 vs. corresponding sham; ^ *p* < 0.05 vs. ADAM17flox IR; *n* = 5 in each group.

Vascular capillary degeneration is a central feature of ischemic retinopathies including diabetic retinopathy and can be reproduced in a mouse model of IR [9,13]. Accelerated death of retinal endothelial cells in the diabetic retina precedes acellular capillary formation and eventually leads to capillary non-perfusion and retinal ischemia [37]. Therefore, next we determined whether conditional inactivation of ADAM17 in endothelial cells affects degenerate capillary formation in the IR-injured retina. As demonstrated in Figure 5A,B, IR caused a significant increase in the number of acellular capillaries in the injured retinas of control ADAM17flox mice 14 days after IR (*p* < 0.01 compared to corresponding sham). Conditional inactivation of ADAM17 markedly reduced acellular capillaries formation in IR-injured retinas of ADAM17Cre-flox mice (*p* < 0.001 compared to ADAM17flox IR; Figure 5A,B).

### 2.6. Lack of Endothelial ADAM17 Reduces Retinal Apoptosis Following IR Injury

Retinal neurovascular degeneration is associated with apoptosis of neuronal and capillary cells following IR injury [9,13]. To assess retinal apoptosis, we performed terminal deoxynucleotidyl transferase dUTP nick end labeling (TUNEL) assay of retinal cryosections. IR (48 h) resulted in marked increase in TUNEL-positive cells in retinas of ADAM17flox mice compared to sham-treated eyes (Figure 5C). Elimination of ADAM17 in the vascular endothelium protected IR-injured retinas as demonstrated by reduction of TUNEL-positive cells in ischemic eyes of ADAM17Cre-flox mice (Figure 5C). We also analyzed the expression of 17–19 kDa active form of caspase-3, an early marker of cellular apoptosis. As expected, in IR-injured retinas of ADAM17flox mice, the levels of cleaved caspase-3 were significantly elevated compared to sham-treated eyes (*p* < 0.05; Figure 5D). Genetic knockdown of endothelial ADAM17 protected retinas from apoptotic cell death as indicated by decreased levels of the active caspase-3 in IR retinas of ADAM17Cre-flox mice (Figure 5D).

### 2.7. Lack of Endothelial ADAM17 Attenuates IR-Induced Oxidative Stress

**Figure 6:**
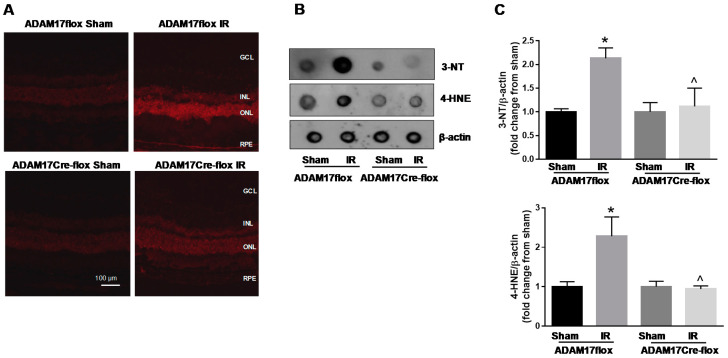
IR-induced oxidative stress is reduced in mice with conditional inactivation of endothelial ADAM17. (**A**) Representative images of DHE-stained retinal sections from control ADAM17flox and ADAM17Cre-flox mice 6 h after ischemic insult. *n* = 3. Scale bar, 100 µm (**B**) Representative dot blot analysis of 4-HNE (4-hydroxynonenal) and 3-NT (3-nitrotyrosine) in retinal tissue from control ADAM17flox and ADAM17Cre-flox mice 6 h after ischemic insult. (**C**) Quantification of optical density of 3-NT and 4-HNE immunoblotting normalized versus actin. Values are mean ± SE. Results are presented as fold change from sham. * *p* < 0.001 vs. corresponding sham for 3-NT and * *p* < 0.05 vs. corresponding sham for 4-HNE; ^ *p* < 0.05 vs. ADAM17flox IR; *n* = 5 in each group.

The generation of excessive reactive oxygen and nitrogen species (ROS and RNS, respectively) is a critical primary event mediating retinal IR-induced injury [13,38]. To understand whether the protective effects of loss of endothelial ADAM17 in the IR-injured retina are associated with reduction in oxidative stress, we compared intracellular superoxide formation in retinal cryosections of ADAM17flox and ADAM17Cre-flox mice using cell-permeable redox-sensitive probe dihydroethidium (DHE). The retinas from sham-operated ADAM17flox and ADAM17Cre-flox mice exhibited low levels of detectable ROS (Figure 6A). Following 6 h of reperfusion, DHE fluorescence increased in retinas of ADAM17flox mice and this effect of IR was significantly reduced in ADAM17Cre-flox mice indicating an attenuation of retinal superoxide levels in these mice (Figure 6A). Overproduction of ROS and RNS can lead to modifications of cellular molecules resulting in formation of 3-nitrotyrosine (3-NT) and 4-hydroxynonenal (4-HNE). Dot blot analysis showed a marked increase of both 3-NT and 4-HNE in IR-injured retinas of ADAM17flox mice 6 h after reperfusion (Figure 6B,C). Endothelial ADAM17 knockdown prevented the increase of both these markers in injured retinas of ADAM17Cre-flox mice (Figure 6B,C).

### 2.8. Knockdown of Endothelial ADAM17 Modulates Inflammatory Responses Following IR

**Figure 7:**
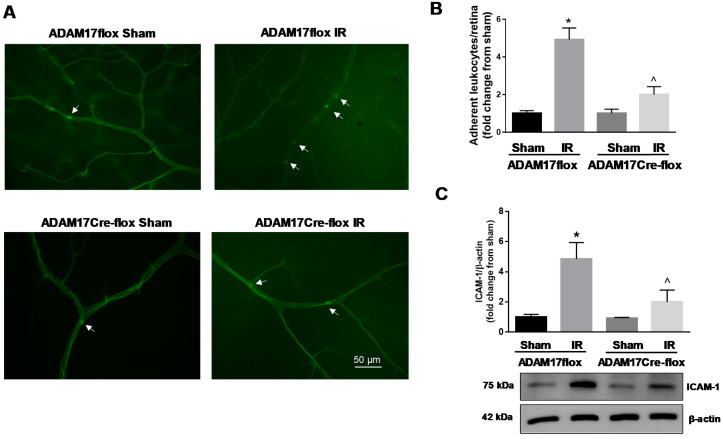
IR-induced retinal leukostasis is reduced in mice with conditional inactivation of ADAM17 in vascular endothelium. (**A**) Representative images of flat-mounted retinas from control ADAM17flox and ADAM17Cre-flox mice 48 h after reperfusion stained with ConA to identify leukocytes adherent to retinal microvessels (white arrows). Scale bar, 50μm. (**B**) Quantification of adherent leukocytes. Data are expressed as adherent leukocytes per retina and presented as fold change of corresponding controls. Values are mean ± SE. Results are presented as fold of change of sham. * *p* < 0.001 vs. corresponding sham; ^ *p* < 0.01 vs. ADAM17flox IR; *n* = 5 in each group. (**C**) Representative Western blots and densitometric analysis of ICAM-1 evaluated 48 h after reperfusion. Actin was used as an internal control. Values are mean ± SE. Results are presented as fold change from sham. * *p* < 0.01 vs. corresponding sham; ^ *p* < 0.05 vs. ADAM17flox IR; *n* = 5 in each group.

IR is associated with increased inflammatory responses in the retina [9,13,39]. To investigate the contribution of endothelial ADAM17 to vascular inflammation, we assessed leukocyte adhesion in retinas of control mice and mice lacking ADAM17 following in situ labeling with fluorescein-labeled concanavalin A (ConA) lectin [35]. Our analysis showed an increase in leukocyte adhesion in ischemia-injured retinal microvasculature of control ADAM17flox mice (*p* < 0.001 compared to corresponding sham; Figure 7A,B). Knockdown of endothelial ADAM17 significantly decreased the number of adherent leukocytes in retinas of ADAM17Cre-flox mice (*p* < 0.01 compared to ADAM17flox IR; Figure 7A,B). As ICAM-1 is involved in retinal IR injury, we evaluated its protein levels in retinas of control mice and mice lacking ADAM17 following IR. As shown in Figure 7C, IR (48 h) increased protein levels of this adhesion molecule in retinas of ADAM17flox mice (*p* < 0.01 compared to corresponding sham) but knockdown of endothelial ADAM17 markedly reduced retinal ICAM-1 in IR-injured eyes of ADAM17Cre-flox mice (*p* < 0.05 compared to ADAM17flox IR; Figure 7C). These data further confirm reduced inflammatory responses in mice lacking endothelial ADAM17.

### 2.9. Knockdown of Endothelial ADAM17 Reduces Cleavage of p75NTR in the Ischemic Retina

**Figure 8:**
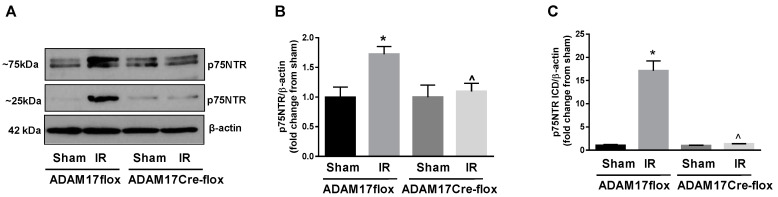
Genetic ablation of endothelial ADAM17 reduces proteolytic processing of p75NTR in IR-injured retinas. (**A**) Representative blots of the full-length (~75 kDa) and cleaved fragment of p75NTR (p75NTR ICD; ~25 kDa) in retinal extracts from control ADAM17flox and ADAM17Cre-flox mice 6 h after reperfusion. (**B**) Densitometric analysis of full-length p75NTR and (**C**) cleaved p75NTR. Actin was used as an internal control. Values are mean ± SE. Results are presented as fold of change of sham. * *p* < 0.05 vs. corresponding sham; ^ *p* < 0.05 vs. ADAM17flox IR; *n* = 5 in each group.

ADAM17-mediated proteolytic cleavage of extracellular domain of p75 neurotrophin receptor (p75NTR) can result in generation of a pro-apoptotic intracellular domain, p75ICD, in a process called regulated intramembrane proteolysis [40,41]. To assess whether this ADAM17-regulated mechanism could contribute to retinal IR-induced cellular degeneration, we assessed cleavage of p75NTR in the IR-injured retinas of control mice and mice lacking expression of ADAM17. Our Western blotting analysis using p75NTR cytoplasmic domain-specific antibody showed that IR (6 h) caused a significant upregulation of p75NTR in control ADAM17flox mice (Figure 8A,B). Consistent with upregulated status of ADAM17 in IR-injured retinas of ADAM17flox mice, increased expression of p75NTR in these mice was associated with accumulation of p75NTR cleaved fragment of about 25 kDa (Figure 8A,C). No changes in the expression of full-length p75NTR or accumulation of its cleaved fragment were observed in mice with conditional inactivation of ADAM17 that undergo IR (Figure 8A–C). These data suggest that ADAM17-mediated p75NTR proteolytic cleavage in IR can contribute to apoptotic events, which culminate in retinal cell degeneration.

## 3. Discussion

Neuronal cell loss and degeneration of vascular capillaries are key features of retinal IR injury. In the current study, we present evidence that inactivation of endothelial ADAM17 activity offers significant protection from retinal IR injury by reducing oxidative stress, alleviating inflammatory responses, and decreasing both neuronal and vascular degeneration. To the best of our knowledge, this study is the first to examine the role of metalloproteinase ADAM17 in IR-induced neuronal and vascular cell damage in the retina.

Retinal ischemia contributes to several vision-threatening pathologies including diabetic retinopathy [1,2,3]. In the ischemic retina, the generation of excessive free radicals can lead to inflammation, vascular leakage, and tissue damage, including both neuronal and capillary degeneration. Since activation of ADAM17 was clearly linked to vascular pathologies as well as inflammatory and neurodegenerative conditions [19,20,21,22,23,30,31,32,33], we were interested in determining whether ADAM17 is involved in neuronal and vascular damage associated with retinal ischemia. Previously, we reported that endothelial ADAM17 activity is implicated in retinal vascular barrier dysfunction in the diabetic retina [35]. In the current study, we confirmed these data by presenting evidence that elimination of endothelial ADAM17 attenuates vascular permeability in IR-injured retinas. Importantly, we demonstrated that inactivation of ADAM17 activity in the endothelium also provides a significant protection against neuronal and vascular damage associated with retinal ischemia-reperfusion. This was evidenced by (1) reduced cell loss in the GCL layer and improved survival of ganglion cells, (2) recovered retinal morphology, and (3) decreased formation of retinal degenerate capillaries in IR-injured retinas of mice lacking endothelial expression of ADAM17. Decreased levels of cleaved caspase-3 and a reduced number of TUNEL-positive cells confirmed that the reduction in neurovascular degenerative processes observed in IR-injured retinas of mice with genetic ablation of endothelial ADAM17 was associated with reduced retinal cell death.

Visual function deficit is among early ocular manifestations in diabetic patients [42,43]. The decline in visual responses including visual acuity and contrast sensitivity was documented in the mouse models of diabetic retinopathy and following retinal IR injury [5,44]. In agreement with these studies, we also detected a significant reduction in visual tracking responses in control mice subjected to retinal IR. Notably, consistent with the protective effects of loss of ADAM17 on neuroretina survival, there was a marked improvement in visual function in endothelial ADAM17 k/o mice subjected to retinal IR.

Excessive production of ROS contributes to neuronal and vascular cell death in the ischemic retina and it was documented that modulating of oxidative stress can protect retinas from IR-induced damage [9,13,38,45]. In agreement with our previous work that demonstrated that endothelial ADAM17 contributes to oxidative stress in the diabetic retina [35], in the current study, we found that inhibition of endothelial ADAM17 activity also markedly attenuates oxidative stress in the IR-injured retina, as evidenced by decreased formation of superoxide (DHE staining) and of oxidative/nitrative stress markers (4-hydroxynonenal/3-nitrotyrosine).

Previous studies have linked upregulation of inflammatory responses in diabetic and IR-injured retinas to the formation of degenerate capillaries [9,11,13]. In the present study, consistent with the ability of ADAM17 to regulate inflammatory responses, we found a reduction in leukocyte adhesion in the ischemic retinas of mice lacking endothelial ADAM17. Leukocyte adhesion is causal to endothelial cell injury and death in the diabetic retina [14]. Accelerated death of retinal endothelial cells in diabetes precedes acellular capillary formation and eventually leads to capillary non-perfusion and retinal ischemia [37]. Therefore, elevated endothelial ADAM17 activity in the IR-injured retina can contribute to acellular capillary formation via the mechanisms promoting leukocyte adhesion. Interestingly, decreased leukocyte adhesion in mice lacking endothelial ADAM17 was associated with the reduction in levels of the leukocyte adhesion molecule ICAM-1. This molecule is a known substrate of ADAM17 in physiological conditions [46]; however, our data suggest that, under ischemic conditions, ADAM17 could be directly involved in controlling ICAM-1 expression through alternate mechanisms that should be further studied. One of such mechanisms could possibly involve ROS-mediated activation of NF-κB [47,48].

The contribution of ADAM17 to apoptotic cell death in the ischemic retina could also be attributed to its activity toward p75 neurotrophin receptor (p75NTR), which has been shown to be involved in regulation of survival and apoptosis of both neurons and endothelial cells [41,49,50,51]. It was reported that the full-length p75NTR undergoes cleavage by ADAM17 to generate a soluble p75NTR extracellular domain [40]. This cleavage event in turn results in generation of a pro-apoptotic intracellular domain as a result of intramembrane proteolysis [40,41]. Previous studies have shown that upregulation of p75NTR expression in retinas in experimental diabetes and in ischemia-reperfusion injury in the brain correlates with accelerated apoptotic death of endothelial cells [51,52]. Our data extend these findings by showing that IR-induced expression of the full-length p75NTR is also associated with increased generation of pro-apoptotic p75ICD fragment. As the reduction in formation of p75ICD in IR-injured retinas of mice lacking endothelial ADAM17 correlates with decreased development of acellular capillaries, we speculate that ADAM17-mediated processing of p75NTR could be involved in endothelial cell injury and death due to IR.

**Figure 9:**
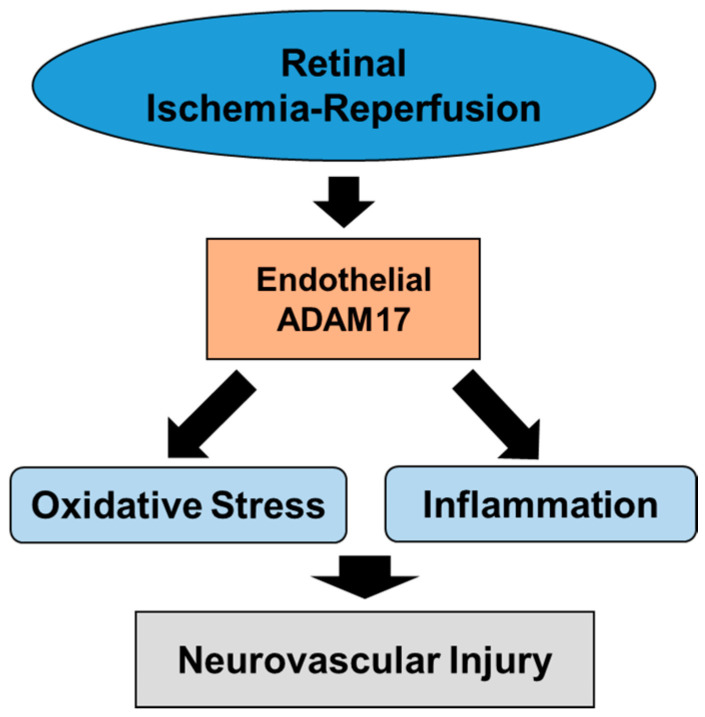
Diagram summarizing the role of endothelial ADAM17 in IR-induced retinal neurovascular damage.

In conclusion, our results provide novel evidence that endothelial ADAM17 activity is an important contributor of IR-induced oxidative stress, inflammation, neurovascular cell death and injury (Figure 9). Finally, our data suggest that interventions directed at regulating ADAM17 activity can be beneficial for preventing and/or alleviating the consequences of ischemia in retinal tissue.

## 4. Materials and Methods

### 4.1. Animals

All the animal procedures were performed in accordance with the statement of the Association for Research in Vision and Ophthalmology (ARVO) for the humane use of animals in vision science and with protocols approved by Augusta University (#2009-0181; approved February 22, 2018). C57Bl/6J mice were purchased from Jackson Laboratories (Bar Harbor, ME, USA). Endothelial-specific ADAM17 knockout (k/o) mice were generated by crossing Adam17tm1.2Bbl/J mice (Stock No: 009597; Jackson Laboratories) which harbor loxP sites flanking exon 2 of ADAM17 with mice expressing Cre recombinase under the control of a Cadh5 promoter (Stock No: 006137; B6.Cg-Tg(Cdh5-cre)7Mlia/J; Jackson Laboratories), as we described before [35]. When bred with ADAM17flox mice, Cre-mediated recombination results in deletion of ADAM17 in the endothelium of developing and quiescent vessels, as well as within a subset of hematopoietic cells [53]. Genotypes of mice were determined by PCR using tail genomic DNA and KAPA Mouse Genotyping Kit (KAPA Biosystem, Wilmington, MA, USA).

### 4.2. Ischemia-Reperfusion Model

Male mice 10–12 weeks of age were subjected to IR in the right eye as described [13,38]. Mice were anesthetized with intraperitoneal injection of ketamine/xylazine (80/12 mg/kg of body weight). Proparacaine (0.5%; Akorn, Lake Forest, IL, USA) was applied to both eyes and tropicamide (1%; Akorn) was used to dilate the pupil. A 32G needle was used to cannulate the anterior chamber and infuse sterile saline. Intraocular pressure was raised to 110 mm Hg, by elevating the saline reservoir, and was maintained for 40 min. Blanching of the retina was monitored to confirm the loss of blood flow. The left eye was treated by briefly inserting a 32G needle into the anterior chamber through the cornea and served as a sham control. Mice were sacrificed at various times after ischemia (6 h, 48 h, 7 days, and 14 days) and eyes were processed for further analysis.

### 4.3. Assessment of Retinal Vascular Permeability

Retinal vascular permeability was assessed by measuring albumin extravasation as we described before [35]. Briefly, 48 h after IR mice were deeply anesthetized with ketamine/xylazine. The chest cavity was opened, and a 20-gauge perfusion cannula was introduced into the left cardiac ventricle. Drainage was achieved by opening the right atrium. The animals were perfused with phosphate-buffered saline (PBS) to rinse out all blood. Retinas then were excised, and serum albumin levels were measured in the perfused retinal tissue by Western blotting analysis using anti-mouse albumin antibody.

### 4.4. ADAM17 Enzymatic Activity

ADAM17 enzymatic activity was measured in retinal extracts from IR-injured and sham-operated mice using SensoLyte Activity Assay kit (AnaSpec, Fremont, CA, USA). This assay employs QXL™520/5-FAM FRET substrate, cleavage of which by active ADAM17 results in increase in fluorescence which is monitored at excitation/emission = 490 nm/520 nm. Retinas were homogenized in assay buffer containing 0.1% (*v/v*) Triton-X 100, incubated for 15 min at 4 °C, and then centrifuged for 15 min at 2000× *g* at 4 °C. ADAM17 activity was normalized to protein concentration. Measurements were made 40 min after incubation with ADAM17 substrate solution as recommended by the manufacturer.

### 4.5. Immunohistochemical Analysis

Immunostaining of retinal sections was performed as described [35]. Mouse eyes were enucleated, embedded in optimal cutting temperature mounting medium (Tissue-Tek, Torrance, CA, USA), frozen on dry ice, and cryostat sectioned (10 µm). Slides were incubated overnight at 4 °C with anti-ADAM17 (1:500; LSBio, Seatle, WA, USA), anti-CD31 (10 µg/mL; R&D Systems, Minneapolis, MN, USA), and anti-RNA binding protein with multiple splicing (RBPMS) (1:500; GeneTex, Zeeland, MI, USA) antibodies, followed by incubation with appropriate fluorescence-conjugated secondary antibodies (Life Technologies, Eugene, OR, USA). Secondary antibody controls (no primary antibody) were included in each experiment. Sections were mounted using Fluoroshield mounting medium containing 4′,6-diamidino-2-phenylindole (DAPI) to visualize nuclei (Sigma-Aldrich, St. Louis, MO, USA) and examined for epifluorescence using a Zeiss Axioplan-2 microscope (Carl Zeiss, Göttingen, Germany) equipped with the Axiovision program (version 4.7; Carl Zeiss).

### 4.6. Analysis of Leukocyte Adhesion

Leukocyte adhesion to the retinal endothelium was evaluated as we described previously [35]. Following the induction of deep anesthesia (ketamine/xylazine, 80 and 12 mg/kg of body weight, respectively), the chest cavity was opened, and a 20-gauge perfusion cannula was introduced into the left ventricle. Drainage was achieved by opening the right atrium. The animals were perfused with 10 mL of warm PBS to wash out non-adherent blood cells. Next, the animals were perfused with 10 mL of fluorescein–labeled concanavalin A (ConA) lectin (40 μg/mL in PBS, pH 7.4; Vector Laboratories, Burlingame, CA, USA) to label the adherent leukocytes and vascular endothelial cells. Residual unbound ConA was removed by perfusion with PBS. The eyeballs were removed and fixed with 4% paraformaldehyde. Retinas were flat-mounted using Fluoromount anti-fading mounting medium (ThermoFisher Scientific, Waltham, MA, USA) and examined for epifluorescence using a Zeiss Axioplan-2 microscope (Carl Zeiss) equipped with the Axiovision program (version 4.7; Carl Zeiss). The total number of adherent leukocytes in the retinal arterioles, venules and capillaries was determined.

### 4.7. Protein Analysis

Proteins were extracted from retinas of sham-operated and IR injured eyes as we described previously [35]. The extracted proteins were quantified (Protein DC Assay; Bio-Rad, Hercules, CA, USA) and subjected to SDS-PAGE. Proteins were transferred to nitrocellulose membranes that were blocked and incubated with primary antibodies against ADAM17 (1:1000; Abclonal, Woburn, MA, USA), albumin (1:10,000; Bethyl Laboratories, Montgomery, TX, USA), cleaved caspase-3 (1:1000; Cell Signaling, Danvers, MA, USA), ICAM-1 (1:500; Abclonal), and p75NTR (1:500; Abclonal) and corresponding horseradish-conjugated secondary antibodies. Actin was used as an internal control (Sigma-Aldrich, St. Louis, MO, USA). Chemiluminescence-based assay was used for protein detection (SuperSignal West Pico Chemiluminescent Substrate; Pierce Biotechnology, Rockford, IL, USA). Scanned images of blots were used to quantify protein expression using NIH ImageJ software (http://rsb.info.nih.gov/ij/).

### 4.8. Dot Blot Analysis

Equivalent amount of proteins prepared from mouse retinal lysates were spotted on nitrocellulose membranes and dried for 5 min at room temperature. The membranes were blocked for 1 h and then probed overnight (4 °C) with either anti-3-nitrotyrosine (3-NT; 1:1000; Cayman, Ann Arbor, MI, USA) or anti-4-hydroxynonenal (4-HNE; 1:1000; Abcam, Cambridge, MA, USA) antibodies. After washing, the membranes were probed with corresponding horseradish peroxidase-conjugated secondary antibody. The immuno-positive spots were visualized by using chemiluminescence-based assay (Bio-Rad, Hercules, CA). Actin was used as loading control. Scanned images of blots were used to quantify protein expression using NIH ImageJ software (http://rsb.info.nih.gov/ij/).

### 4.9. Dihydroethidium (DHE) Staining for Detection of Superoxide

Ten-μm-thick retinal cryosections from retinas of sham-operated and IR-injured eyes were brought to room temperature. Tissue sections were then covered with 2 μM DHE solution (Invitrogen-Molecular Probes, Eugene, OR, USA) and incubated in a light-protected humidified incubator at 37 °C for 20 min. At the end of the incubation, sections were rinsed and mounted using Fluoromount anti-fading mounting medium (Fisher Scientific). The images were taken using a Zeiss Axioplan-2 microscope (Carl Zeiss) equipped with the Axiovision program (version 4.7; Carl Zeiss).

### 4.10. Tdt-dUTP Terminal Nick-End Labeling (TUNEL)

The DNA fragmentation of apoptotic cells in the different experimental groups was analyzed by terminal dUTP nick end labeling (TUNEL) assay (DeadEnd Fluorimetric TUNEL System, Promega, Madison, WI, USA) following the manufacturer’s instructions. The assay measures the fragmented DNA of apoptotic cells by catalytically incorporating fluorescein-12-dUTP at the 3′-OH DNA ends using the enzyme terminal deoxynucleotidyl transferase (TdT). A positive control was set up by treatment of tissue section with DNase (5 U/mL) for 10 min to fragment DNA. Images were obtained using a Zeiss Axioplan 2 fluorescent microscope (Carl Zeiss) equipped with the Axiovision program (version 4.7; Carl Zeiss).

### 4.11. Retinal Vasculature Isolation and Measurement of Acellular Capillaries

Retinal vasculature was isolated at 14 days after IR injury. Freshly enucleated eyes were fixed with 10% buffered formalin for 1 week. Retinal cups were dissected, rinsed, and then incubated with 40 U/mL elastase in 100 mM sodium phosphate buffer, 150 mM sodium chloride, and 5 mM EDTA at pH 6.5 for 12 h [54]. Neural and glial tissues were gently removed and isolated vasculature was stained with periodic acid—Schiff and hematoxylin. Acellular capillaries were counted in six different areas of the mid-retina under the microscope in a double blinded fashion. The number of acellular capillaries was divided by the field area to get number of acellular capillaries per 1 mm^2^ of retina.

### 4.12. Histology and Morphometric Analysis

Retinal morphology was assessed at 7 days post IR-induced injury on hematoxylin and eosin (H&E) stained retinal frozen sections using a Zeiss Axioplan-2 microscope (Carl Zeiss) as previously described [55]. Morphometric analysis included measurements of the thickness of the total retina and inner nuclear layer (INL). The number of cells in the ganglion cell layer (GCL) was quantified by counting cells from the temporal ora serrata to the nasal ora serrata and expressing the data as number of cells per 100 μm length of retina. In addition, the number of ganglion RBPMS-positive cells was quantified. Measurements were made in three adjacent fields (peripheral, mid-peripheral, central) on the temporal and nasal side of the optic nerve at 200- to 300-μm intervals, resulting in a total of six measurements obtained per eye; the initial measurement was made approximately ~200 μm from the optic nerve. Averaged retinal thickness and number of cells in the GCL were presented as percentage compared with the contralateral sham-operated eye.

### 4.13. Optokinetic Tracking for Visual Acuity

Optomotor reflex-based spatial frequency thresholds were analyzed using a visuomotor behavior measuring system (OptoMotry, CerebralMechanics, Lethbridge, AB, Canada) as described [56]. All animals were habituated before the outset of testing with handling and by placing them on the platform for a few minutes at a time. The mice were tested during the first few hours of their daylight cycle. Tracking was defined as a reproducible smooth pursuit with a velocity and direction concordant with the stimulus. Spatial frequency threshold, a measure of visual acuity, was determined automatically with the optokinetic tracking software, which used a staircase paradigm based upon head-tracking movements. Rotation speed (12°/s) and contrast (100%) were kept constant.

### 4.14. Statistical Analysis

Values are mean ± standard error (SE). The data were analyzed by Student’s *t*-test or Mann–Whitney rank sum test using a computer-based software package GraphPad Prism 6.0 (San Diego, CA, USA). For all data, *n* represents the number of animals per group. *p*-values less than 0.05 were considered significant.
